# A rank subdivision of equivalent score for enhancing neuropsychological test norms

**DOI:** 10.1007/s10072-022-06140-6

**Published:** 2022-05-17

**Authors:** Alessio Facchin, Ezia Rizzi, Michela Vezzoli

**Affiliations:** 1 Department of Psychology, University of Milano Bicocca, Piazza dell’ateneo nuovo 1, Milan, Italy; 2 Department of History, Society and Human Studies, University of Salento, Lecce, Italy

**Keywords:** Psychometrics, Neuropsychological tests, Statistics, Nonparametric, Classification

## Abstract

**Introduction:**

Neuropsychological assessment of cognitive functioning is a crucial part of clinical care: diagnosis, treatment planning, treatment evaluation, research, and prediction of long-term outcomes. The Equivalent Score (ES) method is used to score numerous neuropsychological tests. The ES0 and the ES4 are defined respectively by the outer tolerance limit and the median. The intermediate ESs are commonly calculated using a z-score approach even when the distribution of neuropsychological data is typically non-parametric. To calculate more accurate ESs, we propose that the intermediate ESs need to be calculated based on a non-parametric rank subdivision of the distribution of the adjusted scores.

**Material and methods:**

We make three simulations to explain the differences between the classical z-score approach, the rank-based approach, and the direct subdivision of the dependent variable.

**Results:**

The results show that the rank procedure permits dividing the region between ES0 and ES4 into three areas with the same density. The z-score procedure is quite similar to the direct subdivision of the dependent variable and different from the rank subdivision.

**Conclusions:**

By subdividing intermediate ESs using the rank-subdivision, neuropsychological tests can be scored more accurately, also considering that the two essential points for diagnosis (*ES* = 0 and *ES* = 4) remain the same. Future normative data definition should consider the best procedure for scoring with ES.

## Introduction

Neuropsychological assessment of cognitive functioning is a key part of clinical care: diagnosis and treatment planning, treatment evaluation and research [1], prediction of long-term outcomes [2], the individual ability to perform activities of daily living [3], and effects of neurosurgery [4]. Based on clinical history, observations, initial medical history, and behavioural aspects, it is possible to choose neuropsychological tests to employ for each patient. Neuropsychological tests must be psychometrically sound to adequately identify a deficit, accompanied by well-defined standard procedures and accurate normative data [5]. The quantitative evaluation of individuals’ neuropsychological functioning via psychometric tests requires the raw scores to be scored based on the performance of a representative population. In this way, we can compare the performance of a single individual against the performance of a population with similar demographic characteristics (age, education, and gender).

To standardize scores, normative values must be derived from a sample of healthy individuals [6]. Many standardization methods have been proposed to establish normative values, and many of them rely on a non-parametric approach [6]. Neuropsychological data may be affected by ceiling/floor effects and high inter-individual variability. Therefore they do not generally conform to distributional assumptions [7, 8]. For this reason, non-parametric approaches should be preferred when drawing norms. The most well-known non-parametric approach is the Equivalent Score (ES) method [9]. The ES method represents regression-based approaches to standardize neuropsychological tests by non-parametrically drawing cutoffs and controlling for inferential errors [10]. The overall procedure to extract the ESs for a test comprises several steps. The first one entails examining an undivided, demographically composite sample, and calculating the contribution of the demographic variables through multiple regression. Then the original scores have to be adjusted by adding/subtracting that contribution. This permits obtaining test norms based on a relatively small number of participants, in the order of hundreds, compared to the thousands required for covering samples of any age, education, and gender group.

The second step requires deciding with controlled risk whether an adjusted score passes a specific threshold that can be determined using the non-parametric tolerance limits (npTL). Two limits have to be set from the sampled adjusted scores distribution. The outer tolerance limit (OTL) is the cutoff that guarantees (with 95% probability) that no more than 5% of the reference population score is actually below it. The inner tolerance limit (ITL) is the cutoff that guarantees (again with 95% probability) that no less than 5% of the reference population score below it. Therefore, a diagnosis of pathology is rather safe if the score is below the OTL, and a diagnosis of normality is also rather safe if the score is above the ITL. The area between the two limits represents the uncertainty of the classification accuracy.

Finally, starting from the npTLs, the ESs can be determined. The ESs represents an ordinal five-point scale that maps percentile ranks of adjusted scores of a test [9]. As the nature of ESs is non-parametric, they do not depend on the test scores dispersion. As such, ESs possess the properties of the ordinal scale. The ESs permit drawing clinical judgements of the score obtained from a test: ESs equal to 0 and 1 meaning *defective* and *borderline*, respectively; ES equals to 2 meaning *low-end normal* and ESs equal to 3 and 4 meaning *normal*. The OTL represents the limit of the ES equal to 0; the median value of the test score distribution corresponds to the limit of the ES equal to 4. These are the two fixed points in the ES computation. The ESs that lie between ES0 and 4 can be identified by subdividing the range of adjusted scores between the OTL and the median.

To determine the intermediate limits — ES from 1 to 3 — practitioners have mostly relied on a parametric model (i.e. a z-score-based approach) because of the “possible normality of the underlying ability” [7–9]. However, relying on this approach may be problematic. Indeed, determining the intermediate ESs using a parametric approach creates a methodological inconsistency with the non-parametric approach used to determine the two fixed ESs: As explained, the ES0 and ES4 are derived using the OTL and the median of the distribution, both of which are determined using distribution ranks. Additionally, and most importantly, even though we agree that test scores could be expected to be normally shaped in the population, they are usually not normally distributed in normative samples. Certainly, partialling out the effects of demographic characteristics through regression analysis permits obtaining scores (i.e. adjusted scores) that do not have nonlinearity issues. However, adjusted scores might still be non-normally distributed [11, 12]. For example, some tests show a ceiling effect regardless of the demographic variables [11, 13]. Furthermore, the rank-based subdivision was the rule for identifying test cutoffs when TLs and ES methods were unavailable. In a review of normative neuropsychological studies, the authors divided the percentile distribution (from the 5th to the 50th) into three equal parts using percentile intervals [12].

In this paper, we propose that the calculation of the intermediate ESs should be based on a non-parametric rank subdivision of the adjusted scores distribution instead of a parametric distribution model (i.e. z-score). The advantage of the proposed approach is that partitioning the score distribution into three equal parts is performed regardless of the shape of the distribution, like those from a test’s error score. In this way, the three intermediate ESs have the same density, and consequently, the rank classification is more accurate. Furthermore, this method of rank subdivision permits defining each ES scale step as what it is: a rank scale. This paper aims to provide evidence of the good performance of the non-parametric approach to establishing intermediate ESs. To do this, we performed three simulation studies in which different score distributions were considered (i.e. non-parametric vs parametric). To further explore the rank-based approach, we also compared it with another simple approach that could be used to define the intermediate ESs which is based on the direct subdivision of the dependent variable into three equal groups from the OTL to the median. In the case of parametric distribution, this approach should correspond to the z-score subdivision. However, compared to the z-score approach, it is much simpler to implement. Finally, a guide for calculating the TLs and ESs and the R script to implement them is provided.

## Methods

Three simulations were performed. In all the simulations, OTL and ITL were assessed using the non-parametric approach (i.e. npTL), while the intermediate ESs were computed through the z-score approach, the rank subdivision approach, and the subdivision approach based on the value of the dependent variable. The normality of the distributions was checked using the Shapiro–Wilk test.

In the first simulation (i.e. simulation A), we created a sample of 300 not-normally distributed scores. In this sample, the scores represent a test in which the lower the score, the better the performance, such as the execution time of a performance test [14] or a reaction time test [11]. Thus, the npTLs are one-sided in the right part of the distribution.

Like simulation A, simulation B was conducted on a sample of 300 not-normally distributed scores. However, in this simulation, the scores represent a test in which the higher score represents better performance, such as a memory recall test [15–17]. As a consequence, the npTLs are one-sided in the left part of the distribution.

Compared to the other simulations, simulation C was conducted on a sample of 1000 normally distributed scores, where the npTLs are one-sided in the left part of the distribution.

While the calculation of the ESs using a z-score-based approach is well described in the R script provided in [7], the R script for calculating ESs using the rank subdivision approach is available at https://osf.io/v28x6/. This includes both commands for reporting the ES values and the observation number of the ESs. The interested users can easily adapt the R script by simply assigning the adjusted score of the target test to the variable “x” (see the R script). Once they adapt and run the script, the rank of the target observations, the ITL, the OTL, and the 4 cutoffs between the ES0 to ES4 is computed and reported. In addition to reporting the rank positions, ES points are accompanied by corresponding values for the tests. Statistical analyses and figures were performed with R statistical environment [18]. The calculation of TLs was performed using the *tolerance* package [19].

## Results

### Simulation A

**Fig. 1:**
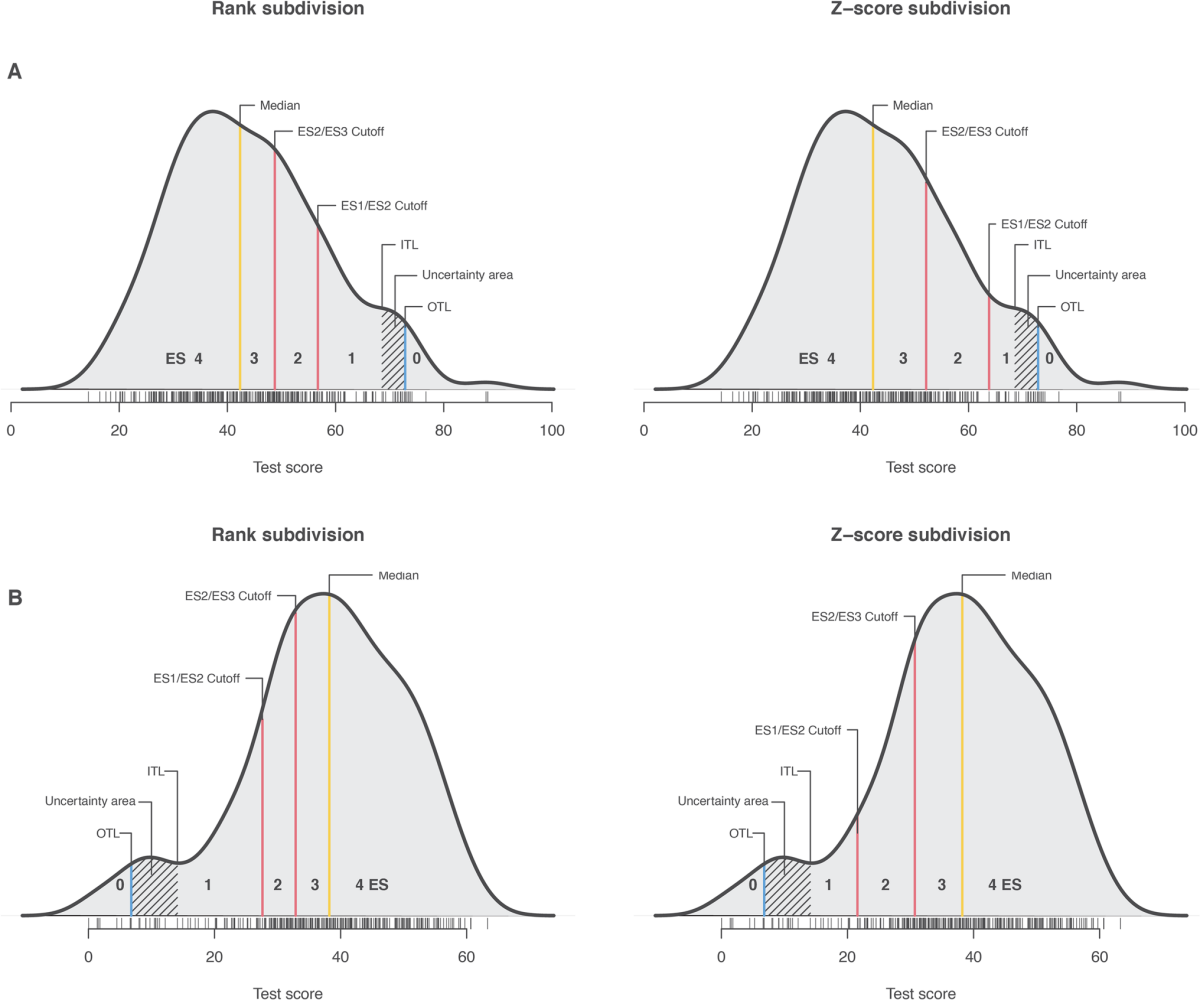
Difference between the rank subdivision and z-score subdivision for the calculation of intermediate ESs. Notes: The reported data represent two examples of typical scoring in which (**A**) lower is better (e.g. task execution time) and (**B**) higher is better (e.g. memory recall task). The distribution represents simulated data from the R code reported online; see text for details. The shaded area in the ES1 area represents the uncertainty of classification between OTL and ITL

The score distribution is not normally distributed (*W* = 0.98 *p* < 0.001), the median is represented by the 151st observation, the ITL by the 280th, and the OTL by the 292nd. As Fig. 1 shows (upper panel), the cutoff points of the intermediate ESs (i.e. ES1, 2, and 3) assume different values in the two approaches: the cutoff scores in the z-score approach are the 221st and 269th observations, while the cutoff scores in the rank subdivision approach are the 198th and 245th observations. The main difference between the rank and z-score approaches lies in the width of the intervals within ES1 and ES3 (Table 1). In the rank-based approach, the width of the intervals among the ESs cutoffs on the dependent variable is different. Still, they have equal density because the underlying rank division is the same. On the contrary, the z-score approach shows a similar size of steps on the dependent variable, but it relies on different underlying population densities. Finally, the direct subdivision of the dependent variable (i.e. the third approach we tested) is between 42.35 (151st observation) and 72.86 (292nd observation). The calculation reports the two cutoffs at 52.52 and 62.69, which correspond (at the nearest observation) to the 223rd and 268th observations. The complex z-score subdivision corresponds in this case (as visible in Fig. 1) to the simple subdivision based on the dependent variable score.

**Table 1:** Comparison of the ESs observation cutoff and population density between ESs among the three approaches (simulation A; *N* = 300)

Method	ES3-ES2 observation rank	ES2-ES1 observation rank	ES3 density *n* (%)	ES2 density *n* (%)	ES1 density *n* (%)
Z-score	221	269	70 (23.33%)	48 (16%)	23 (7.67%)
Rank	198	245	47 (15.67%)	47 (15.67%)	47 (15.67%)
Dependent variable	223	268	72 (24%)	45 (15%)	24 (8%)

### Simulation B

In this second simulation, the score distribution was non-parametric (*W* = 0.97 *p* < 0.001), the OTL is represented by the 9th observation, the ITL by the 21st, and the median by the 151^st^. The cutoff points between ES1 and ES2 and between ES2 and ES3 are the 32nd and 80th observations using the z-score approach. In the rank-based approach, the cutoffs were the 56th and the 103rd. In this case, the cutoff points of the direct subdivision of the dependent variable are 6.79 (9th observation) and 38.21 (151st observation). The calculation reports the two cutoffs at 17.26 and 27.73, which correspond (at the nearest observation) at 26th and 56th observations. The three approaches provide three different results in this simulation because they rely on different calculations.

The results of the z-score and rank-based approaches are comparable to those obtained in the first simulation, except for the slight difference with the median taken (151st in descending order versus ascending order). Conversely, the results of the score subdivision based on the dependent variable distribution are different in the two simulations because the cutoff values depend strictly on the shape and steepness of the distribution in which the ESs fall (Table 2).

**Table 2:** Comparison of the ESs observation cutoff and population density between ESs among the three approaches (simulation B; *N* = 300)

Method	ES1-ES2 observation rank	ES2-ES3 observation rank	ES1 density *n* (%)	ES2 density *n* (%)	ES3 density *n* (%)
Z-score	32	80	23 (7.67%)	48 (16%)	71 (23.66%)
Rank	56	103	47 (15.67%)	47 (15.67%)	48 (16%)
Dependent variable	26	56	17 (5.67)	30 (10%)	95 (31.67%)

### Simulation C

In the third scenario, the score distribution was set to be normal (*W* = 1, *p* = 0.99). Coherently with the chosen distribution, the parametric tolerance intervals were also calculated. Using a non-parametric calculation of the TLs, the OTL is represented by the 39th observation and the ITL by the 62nd observation. Their scores were 32.37 and 34.61, respectively. The parametric calculation of the one side 95% TL with 95% CI was 32.82 and 34.41, respectively. The z-score approach places the intermediate ESs cutoffs at 120th and 278th observations, while the rank-based approach at the 193rd and 347th observations. The direct subdivision of the dependent variable gives 38.25 and 44.13, which correspond to the 120th and 279th observations.

In the case of the normal distribution, the z-score and the subdivision based on the dependent variable score, as expected, give the same results. Conversely, the rank-based score subdivision gives three blocks of the same density of cases. The parametric calculation of TLs, in the case of normally distributed data, is quite similar to the non-parametric one (Table 3).

**Table 3:** Comparison of the ESs observation cutoff and population density between ESs among the three approaches (simulation C; *N* = 1000)

Method	ES1-ES2 observation rank	ES2-ES3 observation rank	ES1 density *n* (%)	ES2 density *n* (%)	ES3 density *n* (%)
Z-score	120	278	81 (8.1%)	158 (15.8%)	223 (22.3%)
Rank	193	347	154 (15.4%)	154 (15.4%)	154 (15.4%)
Dependent variable	120	279	81 (8.1%)	159 (15.9%)	224 (22.4%)

Since a large part of the sample size is between 100 and 600 healthy controls participants [12], a summary of the rank of the OTL, ITL together with ES1-ES2, ES2-ES3, and the median is provided in Table 4. In case of a larger number of participants or different sample sizes, the R scripts we provided compute both the ranks and score values for the target sample size.

**Table 4:** Summary rank observations that correspond to the OTL, ITL, Rank subdivision of ES1-2 and ES2-3, and median

Sample size	OTL	ITL	1ES-2ES	2ES-3ES	Median
100	2	9	18	34	51
125	3	10	23	43	63
150	3	12	27	51	76
175	4	14	32	60	88
200	5	15	37	69	101
225	6	17	41	76	225
250	7	18	46	85	126
275	8	20	51	94	138
300	9	21	56	103	151
325	10	23	61	112	163
350	11	24	66	121	176
375	12	26	70	128	188
400	13	27	75	137	201
425	14	29	80	146	213
450	15	30	85	155	226
475	16	32	90	164	238
500	17	33	95	173	251
525	18	35	99	180	263
550	19	36	104	189	276
575	20	38	109	198	288
600	21	39	114	207	301

Those unfamiliar with the R environment can use the rank values reported in this table to determine the cutoff values

## Discussion

In this paper, we aim to refine the method of calculating the ESs of neuropsychological tests. We provided the rationale for using the rank subdivision of ES score to enhance neuropsychological test norms. The provided simulation examples, with different sample sizes and distributions, showed that the rank subdivision was able (by definition) to resist skewed data. The other two approaches (i.e. the z-score and dependent variable score) give similar results when the adjusted score is normally distributed but different when the data are non-normally distributed.

The use of rank subdivision was motivated by its definition “Equivalent scores are based on the ranks of the adjusted scores; their nature is basically non-parametric, and does not depend on the test scores dispersion” (Capitani & Laiacona, 2017, p. 1224) [9]. This is in contrast with the use of the z-score approach used to define intermediate ESs. Different distributions lead to different results. To the best of our knowledge, the most appropriate solution remains the rank ES: for its simplicity, and because it closely follows the definition of ES, which is an “ordinal five-point scale that maps percentile ranks of adjusted scores of a test” (Capitani & Laiacona, 2017, p. 1223) [9]. The second advantage of rank-based ES is the resulting direct rank subdivision based on population density. There are a number of old and new pieces of evidence that the z-score subdivision does not produce an equal density subdivision in intermediate ES and consequently does not follow perfectly the ranking definition provided above [21–23].

However, rank-based ES scores have some clinical implications, both positive and negative. Given that our proposed approach provides a more accurate subdivision of the ES1-ES3 values based on sample density, it has the main advantage of enhancing the accuracy of diagnosis. As can be seen from Fig. 1, the z-score approach yielded a narrower ES1 width than the rank approach. Compared to the z-based approach, it will be easier to find more patients that fall into the ES1 and fewer in the ES2 with the rank-based approach. We recognize that classification based on these two approaches could change. Nevertheless, the clinical implications of this difference are minimal since the two most important points for the diagnosis remain unchanged: the ES0 and the ES4.

According to Capitani and Laiacona [20], the ES calculation (using the z-score method) also produces the same interval on the *z*-axis. Therefore, a simple and similar method for defining ES1-ES3 based on the direct subdivision of the dependent variable was tested. The results show that the two procedures overlapped perfectly when the data had a normal distribution. When the data are non-parametric, the similarity between these two methods depends only on the shape of the distribution. In simulation A, an approximate result was obtained, but the results were different in simulation B. For those unfamiliar with statistics, the cutoff definition based on the direct subdivision of the dependent variable is the simplest method. Furthermore, to avoid the calculation of rank ES cutoffs, we provide a simple table that reports all rank observations points.

One of the advantages of using ES is its intrinsic ease of interpretation and the fact that it is used in a wide range of neuropsychological tests. Using this approach, a full neuropsychological evaluation may be scored with a single scoring system and test results may be compared easily to identify specific deficits. However, the ES method presents some limitations. The few steps of ES have the disadvantage of including an extensive range of accurate scores. Consequently, it is difficult to appreciate small differences in the case of the evaluation of rehabilitation. Secondly, we are aware that the more critical point for defining a pathological score is the OTL and the correspondent ES0. The intermediate ESs are aleatory, even more so with the procedure listed in this study. The two approaches for calculating ESs (i.e. z-score and rank) will need to be detailed in future normative data studies.

Whether a test has a double scoring system: percentiles and ES, there is the possibility of choosing one over the other according to the situation at hand. For example, a clinician could use ES for reporting a clinical evaluation of a patient, but the same clinician could use percentile for rehabilitation evaluation [6]. In the case of bilateral scoring, i.e. lateralized scores, such as those used for spatial neglect evaluation, ES scoring could not be applied. In these cases, the 95% TL with 95% CI remain valid (OTL and ITL), but they need to be calculated on two sides, obtaining only two cutoffs (i.e. left and right) without using ES scoring [11, 23–25].

In summary, subdividing intermediate ESs using the here so-called rank-subdivision of the distribution of the scores can improve the scoring of neuropsychological tests. Thus, future research aiming at scoring normative data should consider the best procedures at the very least.
